# A Manual of Surgery

**Published:** 1899-06

**Authors:** 


**A Manual of Surgery.** By William Rose, M.B., and Albert Carless. Pp. 1162. London: Baillière, Tindall and Cox. 1898.

We have reviewed this book with much pleasure. It is an excellent work, of the type of small condensed surgical books now prevalent. The descriptions of surgical diseases and operations are well up to date and leave little room for criticism, which would be invidious upon a good book by experienced surgeons. Although we question the propriety of the regular administration of surgical knowledge in concentrated form, this book may be safely recommended. The illustrations are largely borrowed from Tillmanns. The printing and paper are good, and there is evidence of very careful proof-reading.

